# Extemporaneous Preparation of 20 mg/mL Ganciclovir in Artificial Tears in Comparison with Sterile Water for Ophthalmic Administration: Formulation and Stability Study

**DOI:** 10.3390/pharmaceutics15010208

**Published:** 2023-01-06

**Authors:** Jiraporn Leanpolchareanchai, Patamaporn Tangteerakoon, Patcharin Supapsophon, Somsiri Sukavatcharin, Pornchai Simaroj, Jiraphong Suksiriworapong

**Affiliations:** 1 Department of Pharmacy, Faculty of Pharmacy, Mahidol University, Bangkok 10400, Thailand; 2 Chemotherapy Pharmacy Unit, Drug Compounding Section, Pharmacy Department, Faculty of Medicine, Ramathibodi Hospital, Bangkok 10400, Thailand; 3 Pharmacy Services, Somdech Phra Debaratana Medical Center, Faculty of Medicine, Ramathibodi Hospital, Bangkok 10400, Thailand; 4 Department of Ophthalmology, Faculty of Medicine, Ramathibodi Hospital, Mahidol University, Bangkok 10400, Thailand

**Keywords:** ganciclovir, extemporaneous preparation, eye drop, stability, artificial tear

## Abstract

Ganciclovir is available as a lyophilized powder for reconstitution and is normally used to treat ophthalmic viral infections. The use of ganciclovir in artificial tears containing hydrocolloid polymers may prove beneficial to patients during drug application, by prolonging contact time and providing a moistening effect. Therefore, this study aimed to extemporaneously prepare 20 mg/mL ganciclovir in artificial tears and compare its stability with that of a similar concentration of ganciclovir in sterile water (SWI) for ophthalmic administration. First, a compatibility study of the drug with commercial artificial tears found that it was compatible with artificial tears containing sodium hyaluronate (HYA). Subsequently, ganciclovir/0.1% HYA (HYA0.1) and ganciclovir/SWI eyedrops (EDs) in low-density polyethylene (LDPE) eyedrop bottles packed in light-shielded zipper bags were evaluated for their stability at 5 ± 3 °C and 30 ± 2 °C. The results revealed that ganciclovir/SWI ED had good physicochemical and microbiological stability when stored at 5 ± 3 °C for 12 weeks and at 30 ± 2 °C for 8 weeks. Meanwhile, ganciclovir/HYA0.1 ED was stable for 8 weeks when kept at 5 ± 3 °C and at 30 ± 2 °C, but ganciclovir in 0.3% HYA ED could be stored at 5 ± 3 °C for 8 weeks. Nevertheless, particulate matter may need to be investigated using a suitable method to ensure the absence of invisible particles in these preparations. Of these results, ganciclovir/HYA artificial tears and SWI EDs show potential for use as home medications for the treatment of ophthalmic viral infections.

## 1. Introduction

Cytomegalovirus (CMV) is a member of the herpes virus family, and, as with other DNA viruses, life-long latency is a hallmark of CMV infection. CMV anterior uveitis and corneal endotheliitis have been increasingly observed in immunocompetent patients over the past decade [1,2]. Clinical manifestations of CMV corneal endotheliitis include corneal edema, coin-shaped keratic precipitates, elevated intraocular pressure, iritis, and endothelial cell loss [3,4,5,6]. Delays in diagnosis may cause irreversible corneal endothelial decompensation [3], which is vision-threatening. Therefore, appropriate and timely anti-CMV treatment is important.

The mainstay of anti-CMV therapy is ganciclovir and valganciclovir [7]. Ganciclovir is a well-known medication used to suppress the replication of herpes viruses [8]. Systemic ganciclovir, such as intravenous ganciclovir and oral valganciclovir, is recommended for the treatment of CMV anterior uveitis in the initial stage [3,4,5,9]. However, ganciclovir and valganciclovir are expensive and present the risk of systemic side effects, such as granulocytopenia, thrombocytopenia, anemia, azoospermia, and increased serum creatinine levels [10]. In addition, the intravenous or intraocular administration of ganciclovir is usually performed by professional healthcare providers who should be trained in drug administration. Consequently, it cannot be used as a home remedy, as the patients cannot self-administer it, and they are required to be hospitalized for a few weeks to receive it. Therefore, the use of topical ganciclovir therapy, including ophthalmic ganciclovir, has become more popular and is convenient because it produces a more precise effect on the affected area, with fewer systemic side effects [11]. Many studies have confirmed the benefits of topical ganciclovir at concentrations ranging from 1.5 to 20 mg/mL [2,11,12,13,14,15,16]. Only a topical ganciclovir gel with a strength of 1.5 mg/mL is commercially available, and a higher-strength ganciclovir for topical application is inaccessible in many countries [17].

Eye drops are a dosage form that utilizes a noninvasive and patient-friendly technique for administration. Typically, ganciclovir reconstituted in sterile water for injection (SWI) can be instilled into the eyes to treat ophthalmic viral infections. However, the drug solution may cause eye irritation and is rapidly washed away because of the defensive mechanisms of the eyes. Only a few extemporaneous ganciclovir sterile preparations have been reported. One study reported the chemical stability and sterility of frozen ganciclovir (20 mg/mL) solution in normal saline solution (NSS) for 180 days [18]. Frozen ganciclovir was chemically stable and sterile when stored in an amber glass vial at −20 °C for at least 180 days. Another group investigated the stability of 5 and 10 mg/mL ganciclovir eye drops prepared from commercial ganciclovir for infusion (DENOSINE^®^ IV Infusion) and diluted in NSS [19]. Under light-protected conditions at 4 °C and 25 °C, the preparations were physically and chemically stable for up to six weeks. Unfortunately, the drug was unstable when stored at 37 °C even in the second week of the study. However, no sterility tests were performed for these preparations.

Artificial tears are generally composed of hydrocolloid polymers and can moisten the eyes to reduce dryness and irritation [20]. The preparation of ganciclovir in artificial tears may buffer the pH of the drug solution due to the buffering system in artificial tears. Meanwhile, this preparation may simultaneously moisten the eyes during exposure to the drug solution and increase the contact time of the drug. Thus, ganciclovir in artificial tears may increase the effectiveness of the drug in the treatment of ophthalmic viral infections. Additionally, patients can self-medicate at home using a basic ophthalmic administration technique. To the best of our knowledge, no extemporaneous preparation of ganciclovir in artificial tears has been reported. Therefore, this study aimed to extemporaneously compound ganciclovir preparations in multiple-dose artificial tears and determine their stability in comparison with that in SWI. Stability was assessed in terms of physical, chemical, and microbiological aspects at 5 ± 3 °C and 30 ± 2 °C [21,22].

## 2. Materials and Methods

### 2.1. Materials

Lyophilized ganciclovir sodium powder for injection (F. Hoffmann-La Roche Ltd., Basel, Switzerland) and eight commercial multiple-dose artificial tears were purchased from Ramathibodi Hospital, Bangkok, Thailand. The details of eight commercial artificial tears used in this study are described in Table 1. Ganciclovir standard (purity 98% by high-performance liquid chromatography (HPLC)) was purchased from Tokyo Chemical Industry Co., Ltd., Tokyo, Japan. SWI was obtained from General Hospital Products Public Co., Ltd., Pathum Thani, Thailand. HPLC-grade acetonitrile and methanol were obtained from Honeywell Burdick & Jackson™, Honeywell Specialty Chemicals, Singapore. Sodium dihydrogen phosphate (NaH_2_PO_4_) monohydrate, 85% *w/w* ortho-phosphoric acid, and 30% *w/w* hydrogen peroxide (H_2_O_2_) were purchased from Merck KGaA, Darmstadt, Germany. Sodium hydroxide (NaOH) and 37% *w/v* hydrochloric acid (HCl) were obtained from CARLO ERBA Reagents S.A.S., Val de Reuil, France, and RCI Labscan Limited, Samut Sakhon, Thailand, respectively. Fluid thioglycolate medium, tryptic soy broth, and peptone water were purchased from BD Difco™, Becton, Dickinson and Company, Franklin Lakes, NJ, USA.

### 2.2. HPLC Analysis and Validation

HPLC analysis of ganciclovir was modified from the method established in the United States Pharmacopeia (USP) 2022 [23]. Ganciclovir was analyzed using Shimadzu HPLC system (DGU-20A5 degasser, LC-20AD pumping system, SIL-20AHT autosampler, SPD-20A UV/VIS detector; Shimadzu Scientific Instruments, Kyoto, Japan). The drug was eluted through a Hypersil GOLD^TM^ C18 (175 Å, 5 µm, 150 × 4.6 mm, Thermo Fisher Scientific, Waltham, MA, USA) with a guard column (Inertsil^®^ ODS-3, 5 µm, 4.0 × 10 mm, GL Sciences Inc., Tokyo, Japan). A mixture of 25 mM NaH_2_PO_4_ (pH 2.5) and acetonitrile (99:1, *v/v*) was used as eluent at a flow rate of 1 mL/min. The column and sample temperatures were maintained at ambient temperature (25–30 °C). The drug was detected at a wavelength of 254 nm and the injection volume was 20 µL.

The quantitation method was validated according to the International Council for Harmonization (ICH) Harmonized Tripartite Guideline: Validation of analytical procedures: Text and methodology (Q2(R1)) and USP 2022 [23,24]. The requirements for the drug assay are linearity, accuracy, precision, specificity, and limits of detection (LOD) and quantitation (LOQ). Linearity was evaluated over a concentration range of 1–20 μg/mL. Three series of five concentrations of working standard solutions were analyzed by HPLC and used to construct a standard curve. The slope, y-intercept, and regression coefficient (*r*) of the standard curve were calculated. The accuracy test was conducted with three replications of three different spiked standard concentrations (2, 10, and 18 μg/mL), and the %recovery was calculated. Precision was assessed in terms of repeatability and intermediate precision. The repeatability was evaluated by three determinations of three different concentrations (2, 10, and 18 μg/mL) and analyzed within a day, whereas the intermediate precision was determined by three determinations of three different concentrations (2, 10, and 18 μg/mL) of three different analysts. The relative standard deviation (RSD) was calculated as a percentage. The LOD and LOQ were calculated based on signal-to-noise (S/N) ratios of 3 and 10, respectively.

Specificity was evaluated under stress conditions according to a previously reported protocol with some modifications [25,26]. Heat degradation was conducted by incubating the sample at 100 °C for 5 h. For hydrolytic degradation, the standard solution was incubated with an equal volume of either 5 M HCl solution or 5 M NaOH solution. After a 14 h incubation at room temperature, the sample was neutralized prior to HPLC analysis. In the case of oxidation, the drug was mixed with H_2_O_2_ solution to obtain a final concentration of 5% H_2_O_2_ and stored at 80 °C for 2 h. Photolytic degradation was performed, according to a protocol reported elsewhere [26,27] by exposure of the sample to sunlight during the daytime. The exposure time was measured for a total of 96 h when the sample was exposed to sunlight.

### 2.3. Compatibility Test between Ganciclovir and Artificial Tears

Eight multiple-dose artificial tears (HYA0.1, HPMC0.5, HPMC0.3S, DEXH, PEGP, CMCG, CMC, and HPMC0.3A) were selected for this study because they were accessible to the hospital. A certain amount of ganciclovir stock solution (50 mg/mL) was mixed with each artificial tear product to yield 5, 10, and 20 mg/mL ganciclovir. The physical appearance and pH of each artificial tear before and after the addition of the drug were preliminarily determined.

### 2.4. Extemporaneous Preparation of 20 mg/mL Ganciclovir Eye Drops (EDs)

The preparation processes were performed by a well-trained pharmacist using an aseptic technique in a Class III biosafety cabinet at the Chemotherapy Pharmacy Unit, Drug Compounding Section, Pharmacy Department, Faculty of Medicine, Ramathibodi Hospital, Bangkok, Thailand. The standard protocol for compounding sterile preparations of hazardous drugs was applied according to the American Society of Health System Pharmacists (ASHP) guidelines and USP chapters 797 and 800. [23,28] According to the compatibility study, only HYA0.1 artificial tears were compatible with ganciclovir. Therefore, 20 mg/mL ganciclovir EDs were prepared in HYA0.1 artificial tears (ganciclovir/HYA0.1 ED) and compared with SWI used as a vehicle (ganciclovir/SWI ED). In addition, two strengths (0.1% and 0.3%) of HYA artificial tear products are available. The 20 mg/mL ganciclovir in 0.3% HYA artificial tears (ganciclovir/HYA0.3) ED was also prepared and compared with ganciclovir/HYA0.1. Thus, three ED formulations were prepared. The typical reconstitution process was as follows: A vial of lyophilized ganciclovir powder for injection (500 mg) was reconstituted in 10 mL of SWI to obtain a 50 mg/mL ganciclovir solution, which was then mixed thoroughly until the drug was completely dissolved. In this study, low-density polyethylene (LDPE) eyedrop bottles packed in amber plastic zipper bags were used as primary and secondary containers, respectively. The ganciclovir EDs were prepared as described below.

#### 2.4.1. Ganciclovir/HYA0.1 ED

Two milliliters of HYA0.1 artificial tear were drawn from the original LDPE container and then 2 mL of 50 mg/mL ganciclovir reconstituted solution was added into the bottle of HYA0.1 artificial tear containing 3.0 mL of artificial tear. The solution was then mixed gently to obtain 5 mL of 20 mg/mL ganciclovir/HYA0.1 ED.

#### 2.4.2. Ganciclovir/HYA0.3 ED

The extemporaneous preparation of ganciclovir/HYA0.3 ED was performed as previously described for Ganciclovir/HYA0.1 ED.

#### 2.4.3. Ganciclovir/SWI ED

Two milliliters of 50 mg/mL ganciclovir reconstituted solution were added to 3 mL of SWI in an LDPE eye drop bottle and mixed gently. A 20 mg/mL ganciclovir/SWI ED solution was obtained with a final volume of 5 mL.

### 2.5. Physicochemical Evaluation of Extemporaneous 20 mg/mL Ganciclovir EDs

#### 2.5.1. Physical Properties

The physical properties of the preparations were evaluated in terms of their appearance, pH, osmolality, and viscosity. The appearance was visually observed by the naked eye for clarity, precipitates, and gas bubbles. The results for clarity, precipitates, and gas bubbles were estimated and rated on a Likert scale ranging from − (could not be observed) to +++ (clearly observable). The pH and osmolality were measured using a calibrated pH meter (LAQUAtwin pH-22, HORIBA, Ltd., Kyoto, Japan) and osmometer (3250 Single-sample osmometer, Advanced Instruments, Norwood, MA, USA), respectively. The change in pH was considered acceptable if it did not vary by more than one pH unit from the initial value [21]. Viscosity measurements were performed using a rotational rheometer (cone and plate model C60/1, HAAKE™ RotoVisco™, Thermo Fisher Scientific, Karlsruhe, Germany) at a shear rate of 1000 s^−1^ and a temperature of 30.0 ± 0.5 °C.

#### 2.5.2. Drug Content Assay

The drug content was analyzed using HPLC. Drug extraction prior to analysis was performed as follows. A quarter of a milliliter of the formulation was withdrawn from the bottle using a calibrated 1 mL sterile disposable Luer slip syringe. The drug was then extracted with 1:1 *v/v* of methanol and water in a 5 mL volumetric flask using a vortex mixer and sonicated for 15 min. The volume was adjusted to 5 mL using a mixture of methanol and water (1:1, *v/v*). The precipitates were removed by centrifugation at 4200 rpm for 15 min, and 50 µL of the supernatant was diluted and adjusted to a volume of 5 mL using the mobile phase. The sample was filtered through a 0.45 μm nylon syringe filter prior to HPLC analysis. The percent labeled amount of drug was calculated according to Equation (1) and compared with the USP limit [23].

(1) $$\%\;\mathrm{Labeled}\;\mathrm{amount}=\frac{\mathrm{Analyzed}\;\mathrm{concentration}\;\mathrm{of}\;\mathrm{ganciclovir}}{20\;\mathrm{mg}/\mathrm{mL}}\;\times \;100$$

The drug extraction method was verified by spiking an accurate weight of ganciclovir standard into the 20 mg/mL ganciclovir in artificial tears and SWI preparations. The drug was withdrawn and extracted, as described previously. After HPLC analysis, the %recovery was calculated based on the spiked amount of the standard. All experiments were performed in triplicate.

### 2.6. Stability Testing of Extemporaneous 20 mg/mL Ganciclovir EDs

Five bottles each of 20 mg/mL ganciclovir EDs were kept in a refrigerator (5 ± 3 °C) and incubator (30 ± 2 °C) and protected from light using amber zipper bags for at least 12 weeks. At the pre-designed time point, the physical and chemical stabilities of the preparations were tested as follows.

#### 2.6.1. Physical Stability Evaluation

Physical stability of the preparations was evaluated in terms of appearance, pH, osmolality, and viscosity. This evaluation was performed as previously described.

#### 2.6.2. Chemical Stability Evaluation

Ganciclovir content was analyzed by HPLC, as previously described. The percentage of the remaining ganciclovir content was calculated using Equation (2). The baseline concentration (day 0) was defined as 100%, and the subsequent concentration at each time point was calculated as the percentage of remaining drug content compared to the initial concentration. The acceptance criterion for the chemical stability was defined as 90–110% of the baseline concentration (including the limit of the 95% confidence interval of the measures) [21,29].

(2) $$\mathrm{Remaining}\;\mathrm{ganciclovir}\;\mathrm{content}\;(\%)=\frac{\mathrm{Analyzed}\;\mathrm{amount}\;\mathrm{of}\;\mathrm{ganciclovir}\;\mathrm{at}\;\mathrm{each}\;\mathrm{time}\;\mathrm{point}}{\mathrm{Analyzed}\;\mathrm{amount}\;\mathrm{of}\;\mathrm{ganciclovir}\;\mathrm{at}\;\mathrm{initial}\;\mathrm{time}\;}\;\times \;100$$

#### 2.6.3. Microbiological Stability Evaluation

The sterility assay was carried out by the Center of Analysis for Product Quality (CAPQ), Microbiology, Faculty of Pharmacy, Mahidol University, in line with the USP 2022 for <71> Sterility Tests [23]. At the predetermined time point, three bottles of each preparation were used to test sterility using a membrane filtration method. Half of the contents of each bottle were withdrawn and pooled. The pooled sample was filtered through a membrane prewashed with Fluid A and aseptically cut into two equal parts. Each part was directly transferred to a fluid thioglycolate medium and a soybean casein digest medium, which were subsequently incubated at 30–35 °C and 20–25 °C, respectively, for 14 days. The culture medium was carefully examined for microbial growth.

### 2.7. Data Analysis

The results are expressed as the mean ± standard deviation of at least three measurements.

## 3. Results and Discussion

### 3.1. HPLC Analysis and Validation

**Figure 1:**
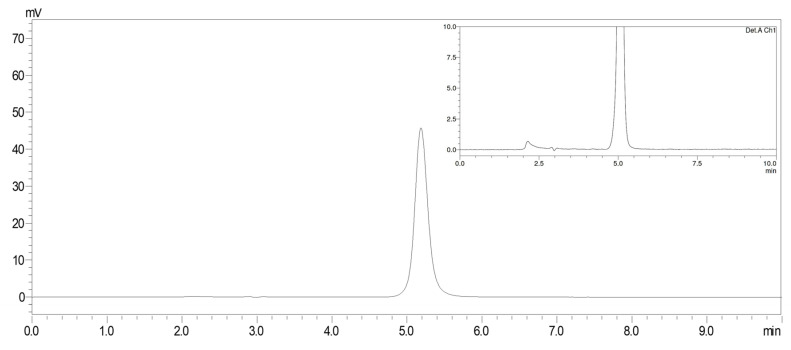
HPLC chromatogram of ganciclovir standard solution at 10 μg/mL under the optimal HPLC condition. An inset illustrates the enlarge view of the chromatogram.

The optimal HPLC composition of ganciclovir was obtained using a mixture of 25 mM NaH_2_PO_4_ (pH 2.5) and acetonitrile (99:1, *v/v*) as the mobile phase. Figure 1 and Table 2 present the HPLC chromatogram and validation results for ganciclovir, respectively. By this condition, ganciclovir standard was eluted at approximately 5.1–5.2 min (RSD 0.4%) with a resolution of 7.4, a tailing factor of 1.1, and a theoretical plate of 4130. These values fell within the limit of system suitability for an assay of ganciclovir for injection in USP 2022 [23]. Regarding the validation results, the system was linear with an *r* value of 0.9998 over a concentration range of 1–20 μg/mL. The repeatability and intermediate precision showed RSD values of 1.2 and 2.0%, respectively. The LOD was 0.03 μg/mL and the LOQ was 0.10 μg/mL. The %recovery was found to be 99.5 ± 1.2 % with RSD of 1.2%. These values are acceptable for the method validation criteria [23,24,30,31].

**Figure 2:**
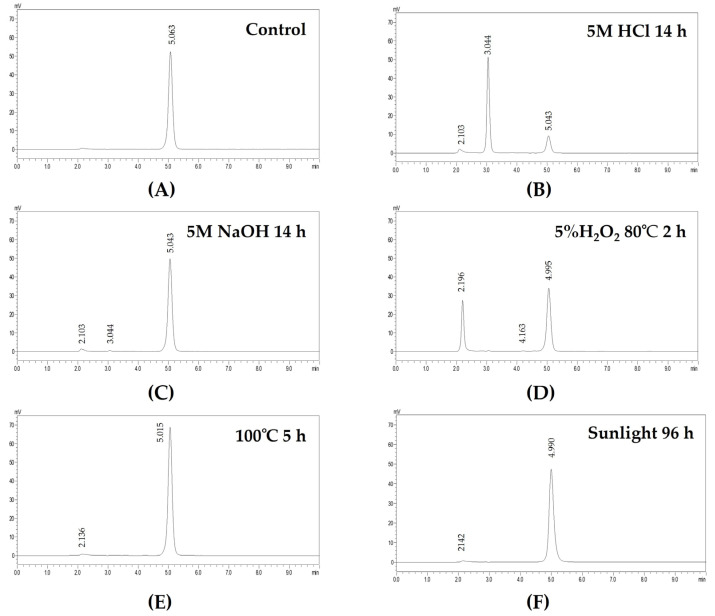
HPLC chromatograms of ganciclovir after being subjected to the stress conditions. (**A**) Control; (**B**) acid hydrolysis; (**C**) base hydrolysis; (**D**) oxidation; (**E**) heat degradation; and (**F**) photolytic decomposition.

Forced degradation testing was performed to check for specificity and stability-indicating properties of the method. The ganciclovir standard was prepared and subsequently subjected to heat, acid, base, oxidation, and daylight stress conditions. Various conditions were employed for the forced degradation of ganciclovir, depending on the method used for detection and the initial amount of the drug [25,32]. Under these conditions, no traces of degradation products were observed because of the different detection methods and initial concentrations of the drug. Therefore, the forced degradation conditions were modified further. As shown in Figure 2, ganciclovir was separately eluted at 5.2 min without any interference from other peaks. Under these forced degradation conditions, ganciclovir is degraded by acid and oxidation. The obtained results agree with those of Ramesh et. al. [25] reporting that there was a slight change in mean peak area under both acidic (2 N HCl) and basic (2 N NaOH) conditions, and there was a significant change in peak area under oxidation (5% H_2_O_2_) conditions. The HPLC chromatograms showed good separation of ganciclovir from other peaks. An enlarged view of the traces of degradation products is shown in Figure S1. The results indicated that the modified HPLC conditions could distinguish the ganciclovir peak from other degraded products and could be used for the stability study of ganciclovir.

The extraction of ganciclovir from the drug preparations was also tested. The results revealed that ganciclovir can be completely extracted from artificial tears. No excipient peaks from the vehicle interfered with the peak of ganciclovir (Figure S2). The percent recovery was found in the range of 96.1–103.6% with a %RSD value of 2.6, which was within the USP acceptable limit [23].

### 3.2. Compatibility of Ganciclovir and Artificial Tears

Artificial tears commonly contain hydrocolloid polymers that maintain the eye moisture. Prior to the addition of ganciclovir to artificial tears, compatibility between the drug and artificial tears must be studied. The eight commercial artificial tears used in this study were CMCG, DEXH, PEGP, HPMC0.3A, CMC, HPMC0.5, HYA0.1, and HPMC0.3S. To determine the effect of drug concentration, the concentration of ganciclovir in the admixture was varied to be 5, 10, and 20 mg/mL. Table 3 shows the compatibility results and the pH values obtained after mixing with the drug. The initial pH of all the artificial tears generally ranged from 6.3–7.9. After mixing, the pH of all admixtures gradually increased with the concentration of ganciclovir and reached approximately 9.1–10.3 at the highest concentration of ganciclovir. Ganciclovir in SWI at concentrations of 5, 10, and 20 mg/mL was used as a control and the pH was almost constant at 10.4–10.5. The optimal pH range of ganciclovir injection after reconstitution at a concentration of 50 mg/mL is 10.8–11.4 as stated in the USP monograph of ganciclovir for injection [23]. Ganciclovir has low solubility in water, and its pKa values are 2.2 and 9.4 [33]. Drug dissolution is required with the aid of sodium hydroxide; thus, the solution for injection has a high alkaline pH to maintain the solubility of the drug.

After mixing and storing at room temperature (30 °C), none of the admixtures precipitated at the lowest ganciclovir concentration. Increasing the concentration of ganciclovir from 5, 10, and 20 mg/mL led to the formation of precipitates in all admixtures, except for DEXH, HPMC0.5, and HYA0.1. However, the drug in the DEXH and HPMC0.5 admixtures precipitated when stored in a refrigerator overnight. From these data, only the HYA0.1 admixture was the most stable and compatible with the ganciclovir solution at a concentration range of 5–20 mg/mL. It was hypothesized that the incompatibility of the drug and artificial tears was associated with the buffer system and hydrophilic polymers of the artificial tears. The stronger buffer system of artificial tears attempted to maintain the pH of the solution and the lower pH of the admixtures, thus inducing the precipitation of the drug. HYA0.1 artificial tears consist of sodium hyaluronate as a hydrophilic polymer and ε-aminocaproic acid as a buffer in the eye [34]. This system may help to prevent drug precipitation. As mentioned above, 20 mg/mL ganciclovir is commonly used to treat CMV. Therefore, we chose 20 mg/mL ganciclovir in HYA0.1 for further study in comparison with this drug in SWI.

### 3.3. Extemporaneous Preparation of 20 mg/mL Ganciclovir/HYA0.1 and SWI EDs

**Figure 3:**
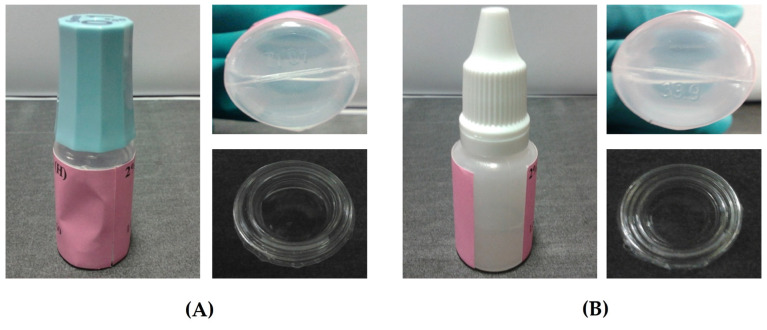
Examples of appearance of (**A**) 20 mg/mL ganciclovir/HYA0.1 ED and (**B**) ganciclovir/SWI ED after preparation.

Extemporaneous ganciclovir/HYA0.1 and ganciclovir/SWI EDs were prepared at 20 mg/mL of the drug. The preparations are shown in Figure 3, and their characteristics are summarized in Table 4. The ganciclovir/HYA0.1 ED was filled in the original container of HYA0.1, whereas the ganciclovir/SWI ED was packaged in an LDPE eyedrop bottle with an outermost amber plastic zipper bag. The solutions of both preparations were clear, colorless, and free of precipitates and bubbles as observed by visual inspection. The pH of the EDs was approximately 10.0–10.5. Typically, human eyes can tolerate a wide pH range of solution over 4.0–10.0 owing to the buffering capacity of tears, which contain multiple buffering components such as bicarbonate and proteins, etc. [35,36]. The osmolarity of normal tears is 300–310 mOsm/L [37]. The average daytime tear osmolality was reported to be in the range of 299–323 mOsm/kg, while another range of tear osmolality was found to be 282–288 mOsm/kg immediately after eye closure for 6–8 h such as in sleep [38,39]. The osmolality of ganciclovir/HYA0.1 ED was 290 mOsm/kg H_2_O, which was close to the normal tear osmolality. However, the osmolality of ganciclovir/SWI ED was >2-fold lower than that of ganciclovir/HYA0.1 ED owing to the absence of tonicity adjusters in the formulation. Despite the lower osmolality of ganciclovir/SWI ED by half that of normal tears, patients could tolerate hypotonic tear preparations and the majority of patients preferred the isotonic preparation [40].

**Figure 4:**
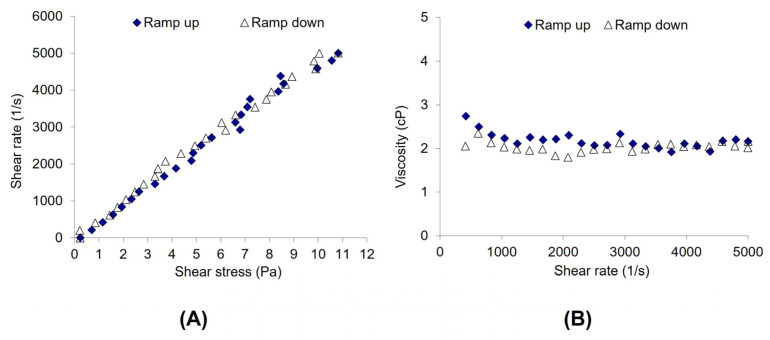
Rheology profiles of ganciclovir/HYA0.1 ED plotted between shear stress vs. shear rate (**A**) and shear rate vs. viscosity (**B**). The viscosity and shear stress were recorded while the shear rate was increasing (blue diamond) and decreasing (blank triangle) over the range of 10–5000 s^−1^.

The viscosity of ganciclovir/HYA0.1 ED was approximately 2.20 cP while that of ganciclovir/SWI ED was 0.93 cP. The former had a higher viscosity value owing to the presence of sodium hyaluronate in the preparation, whereas the latter was close to water. The admixture of ganciclovir in HYA0.1 showed Newtonian rheological behavior (Figure 4) as shear stress linearly increased with the shear rate. HYA artificial tears have been reported to exhibit non-Newtonian (pseudoplastic) behavior [41]. The rheological behavior of ganciclovir/HYA0.1 ED changed from pseudoplastic to Newtonian and was attributable to the dilution effect of the ganciclovir solution, leading to a low concentration of sodium hyaluronate in the preparation. Although the pseudoplastic or shear-thinning flow may provide increased ocular retention time at a low shear rate and comfort to the ocular surface while blinking, the Newtonian flow of the ED may improve the tear film rheological performance of patients. This may smoothly spread the tear film during eye closure. The low viscosity of EDs was below the blur threshold (20–30 cP) [42,43,44] and may not cause excessive blurring after the application of EDs. The drug content in both preparations was in the range of 90–110%, and they passed the sterility test. Both preparations demonstrated good physicochemical and microbiological properties and were further used for stability studies.

### 3.4. Stability Study

The stability of ganciclovir/HYA0.1 ED and ganciclovir/SWI ED was studied at 5 ± 3 °C and 30 ± 2 °C. The physical appearance and sterility results after storage at 5 ± 3 °C and 30 ± 2 °C are summarized in Table 5 and Table 6, respectively. The results revealed that the integrity of both EDs stored under both conditions was completely sealed and comparable to that of the original containers, and no leakage was observed during the entire study period.

**Figure 5:**
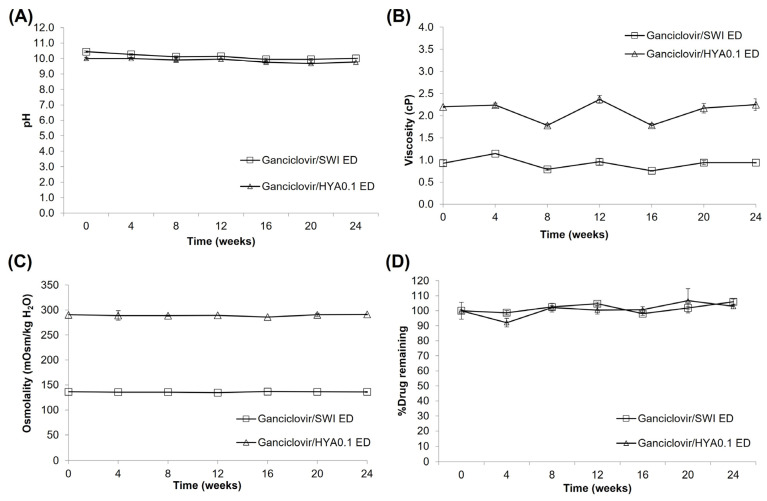
Stability data (pH (**A**), viscosity (**B**), osmolality (**C**), %drug remaining (**D**)) of ganciclovir/HYA0.1 ED and ganciclovir/SWI ED after stored at 5 ± 3 °C for 24 weeks.

Regarding the formulations stored at 5 ± 3°C, both EDs had colorless and clear solutions (Table 5); however, ganciclovir/HYA0.1 ED had needle-like crystals that settled down at the bottom of the bottle when stored for 12–24 weeks. The observed crystals were redissolved by gentle mixing or standing at ambient temperature for 120 min. The crystals were sufficiently large to be detectable by visual inspection at 12 weeks. Nevertheless, very small particles may have been present in the sample before they were observable. Proper and sensitive methods for the inspection of particulate matter may be needed to ensure that preparations are free from unobservable particles over their shelf life. According to the USP monograph [23], two standard methods, namely, light obscuration and microscopy with a standard circular diameter graticule, are suggested. No gas bubbles were detected in ganciclovir/HYA0.1 ED for 24 weeks. Many gas bubbles were observed in the ganciclovir/SWI ED after storage for 16 weeks. The pH of ganciclovir/HYA0.1 ED and ganciclovir/SWI ED slowly decreased from 9.99 to 9.77 and from 10.44 to 10.01, respectively, at the end of the study (Figure 5). The viscosity of ganciclovir/HYA0.1 ED varied from 1.78 to 2.37 cP while that of ganciclovir/SWI ED was in the range of 0.79–1.14 cP. The osmolality of both EDs was almost constant over the period of study and the %drug remaining in both EDs was in the range of 90–110%. Although the presence of crystals was observed in ganciclovir/HYA0.1 ED, after completely dissolving these crystals, the remaining drug content in the preparation was in the range of 90–110%. In addition, no trace of degradation products was observed in the HPLC chromatograms (Figure S3). When testing the sterility of the EDs (Table 5), both passed the sterility test for 12 weeks. The preparations failed the sterility test when stored for 16 weeks. The results indicated that the ganciclovir/SWI ED was physically, chemically, and microbiologically stable at 5 ± 3 °C for 12 weeks in an LDPE eyedrop bottle. The ganciclovir/HYA0.1 ED was physically stable for 8 weeks. Nevertheless, this formulation was chemically and microbiologically stable for 12 weeks. The physical instability of ganciclovir/HYA0.1 ED, as evidenced by the needle-like crystals, could be redissolved by gentle mixing. To ensure the stability of the preparation for patients, the beyond-use date of ganciclovir/HYA0.1 ED should not be beyond 8 weeks. If the crystals occur during storage, the ED should be gently mixed before use until no crystals can be visually observed.

**Figure 6:**
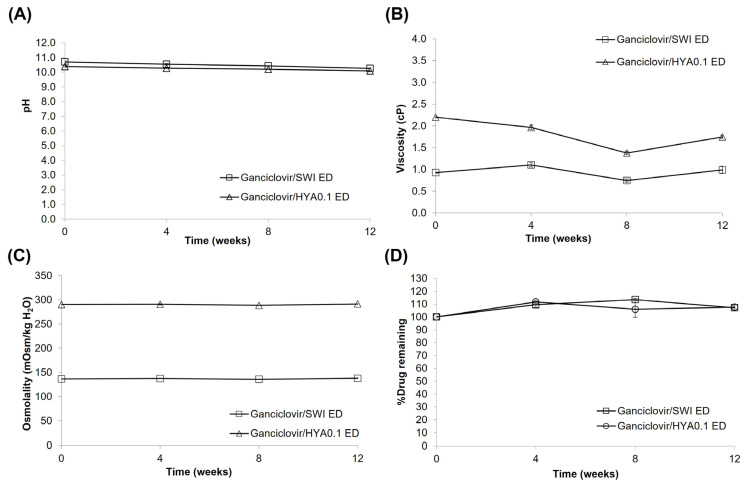
Stability data (pH (**A**), viscosity (**B**), osmolality (**C**), %drug remaining (**D**)) of ganciclovir/HYA0.1 ED and ganciclovir/SWI ED after stored at 30 ± 2 °C for 12 weeks.

When stored at 30 ± 2 °C (Table 6), both preparations were colorless and clear for 8 weeks; however, they became slightly translucent after 12 weeks as observed by the naked eye. To confirm the formation of colloidal-sized crystals, a reliable standard method, such as light obscuration or microscopy, is required for measuring particulate matter. No precipitation at the bottom of the container or gas bubbles were observed for 12 weeks. Again, the pH of ganciclovir/HYA0.1 ED and ganciclovir/SWI ED gradually decreased from 10.69 to 10.26 and 10.39 to 10.08, respectively; however, their osmolality remained almost constant (Figure 6). The viscosity of ganciclovir/SWI ED varied over the range of 0.74–1.11 cP whereas that of ganciclovir/HYA0.1 ED tended to decline over time. The drug content remained over 90%, but not more than 110% relative to the initial content. The HPLC chromatograms did not show any traces of degradation products (Figure S3). Both EDs were confirmed to be sterile for 12 weeks when stored at 30 ± 2 °C (Table 6). The results suggest that ganciclovir/HYA0.1 ED and ganciclovir/SWI ED were physically, chemically, and microbiologically stable at 30 ± 2 °C for 8 weeks.

A previous study reported that ganciclovir solutions at 5 and 10 mg/mL in NSS were chemically stable for 4 weeks or 28 days at 4 °C and −20 °C in PVC bags and 4 °C in an ADFuse infusion system [45]. At week 4, the pH of the solution was maintained at 10.2 ± 0.1. Another report revealed that 5 and 10 mg/mL ganciclovir EDs in NSS were physically and chemically stable at 4 and 25 °C for only six weeks in glass tubes [19]. The precipitates were visualized when storing 5 mg/mL ganciclovir ED at 4 °C and 25 °C for 8 and 12 weeks, respectively, while storing 10 mg/mL ganciclovir ED at both temperatures for 8 weeks. However, the drug content of 10 mg/mL ganciclovir ED declined to less than 90% at week 8 of storage at 25 °C as compared to the day of preparation. The pH values of 5 and 10 mg/mL solutions after 12 weeks of storage at 4 °C were 10.1 and 10.5, respectively. Although the pH was maintained at approximately 10.1–10.5 until the end of the study, precipitation of drug crystals still occurred at the start of week 8. The higher concentration of the drug and lower storage temperature predominantly accelerated crystal formation, whereas the pH of the solution had a minor effect on the precipitation of the drug. The shorter shelf life of the ganciclovir EDs was possibly due to the use of NSS as a diluent in the preparations. Compared with our EDs containing higher drug concentrations, no precipitate was observed in the ganciclovir/SWI ED over the entire study period at 5 ± 3°C. The NSS used in the reported preparation to maintain the isotonicity of the drug solution may accelerate the physical instability of EDs. At 20 mg/mL of ganciclovir/HYA0.1 ED, the drug did not recrystallize when stored at 5 ± 3 °C for eight weeks. The composition of HYA0.1 artificial tears used as a vehicle may not accelerate drug crystallization upon storage at 5 ± 3°C.

### 3.5. Preparation and Stability Study of Ganciclovir/HYA0.3 ED

Because two strengths of HYA artificial tear products containing sodium hyaluronate 0.1% and 0.3% are available in Thailand, we, therefore, further investigated the physical and chemical stability of ganciclovir in HYA0.3% artificial tear (ganciclovir/HYA0.3 ED) and compared it with that of ganciclovir/HYA0.1 ED. The stability of ganciclovir/HYA0.3 ED was studied at temperatures of 5 ± 3 °C and 30 ± 2 °C. The results are summarized in Table 7. Ganciclovir/HYA0.3 ED had initial characteristics similar to ganciclovir/HYA0.1 ED with a pH value of 10.42 ± 0.02, an osmolality of 290 ± 2 mOsm/kg H_2_O, and %labeled amount of 100.4 ± 3.5%. However, ganciclovir/HYA0.3 ED exhibited approximately three times higher viscosity (6.69 ± 0.13 cP) than ganciclovir/HYA0.1 ED (2.20 ± 0.01 cP) due to the three times higher concentration of sodium hyaluronate in the formulation.

Regarding the stability of the preparation (Table 7), the EDs under both conditions retained the complete integrity of the packaging and colorless solution without gas bubbles over the study period. When stored at 5 ± 3 °C for eight weeks, the properties of ganciclovir/HYA0.3 ED remained almost unchanged or comparable to the initial value. However, after eight weeks, needle-like crystals were again observed at the bottom of the container and could be completely dissolved at ambient temperature. The %remaining of the drug was in the range of 90–110% when they were stored for 16 weeks. No trace of degradation products was detected in the chromatograms (Figure S3). However, at week 20 of storage, the containers had %drug remaining lower than 90%. Although the preparation stored at 30 ± 2 °C was physically stable, it was chemically unstable even at the first sampling time point (4 weeks), as the %drug remaining was lower than 90%. Therefore, ganciclovir/HYA0.3 ED was stable for at least eight weeks when stored at 5 ± 3 °C. The preparation could also be used after eight weeks of storage. However, if crystals occur, the preparation should be well mixed prior to use. It is not recommended to use it when stored at 5 ± 3 °C after 16 weeks and to keep the preparation at room temperature. Although this preparation is physically and chemically stable, further studies on sterility are required.

## 4. Conclusions

Extemporaneous preparations of 20 mg/mL ganciclovir EDs were successfully compounded into SWI, HYA0.1, and HYA0.3. It is recommended that ganciclovir/SWI ED in an LDPE eyedrop bottle be stored at 5 ± 3 °C for 12 weeks and at 30 ± 2 °C for 8 weeks after preparation. Meanwhile, ganciclovir/HYA0.1 and ganciclovir/HYA0.3 EDs should be kept at 5 ± 3 °C for eight weeks, but ganciclovir/HYA0.1 ED can be stored at 30 ± 2 °C for eight weeks. In case crystals appear during storage, the preparations should be well mixed until they are completely dissolved before use. However, owing to the limitations of this study, particulate matter with a very small size may need to be investigated using a proper and sensitive method, such as light obscuration or microscopy. Ganciclovir/HYA EDs may benefit patients by prolonging ocular retention and comforting the eyes. These preparations show potential as home medications for the treatment of ophthalmic CMV infections in patients who do not require hospitalization, thus reducing the cost of treatment. Nevertheless, the efficacy and safety of these preparations require further studies in in vivo models or clinical trials.

## Figures and Tables

**Table 1:** A list of commercial artificial tear products used in this study with their source and compositions as stated in the product information or leaflet.

Product Code	Source	Active Ingredients	Inactive Ingredients
CMCG	Allergan Australia Pty Ltd., North Sydney, NSW, Australia	0.5% Carboxymethyl cellulose sodium and 0.9% glycerin	Boric acid, calcium chloride dihydrate, erythritol, levocarnitine, magnesium chloride hexahydrate, potassium chloride, purified water, PURITE^®^ (stabilized oxychloro complex), sodium borate decahydrate, and sodium citrate dihydrate
DEXH	Alcon Laboratories, Puurs-Sint-Amands, Belgium	0.1% Dextran 70 and 0.3% hydroxypropyl methyl cellulose	Disodium edetate, sodium chloride, potassium chloride, benzalkonium chloride, hydrochloric acid and/or sodium hydroxide (to adjust pH), and purified water
PEGP	Alcon Laboratories, Lake Forest, CA, USA	0.4% Polyethylene glycol 400 and 0.3% propylene glycol	Aminomethyl propanol, boric acid, hydroxypropyl guar, POLYQUAD^®^ (polyquarternium-1), potassium chloride, sodium chloride, sorbitol, hydrochloric acid and/or sodium hydroxide (to adjust pH), and purified water
HPMC0.3A	Alcon Laboratories, Fort Worth, TX, USA	0.3% Hydroxypropyl methyl cellulose	Carbopol 980, phosphonic acid, purified water, sodium hydroxide, sodium perborate, and sorbitol
CMC	Allergan Australia Pty Ltd., North Sydney, NSW, Australia	0.5% Carboxymethyl cellulose sodium	Boric acid, calcium chloride, magnesium chloride, potassium chloride, purified water, sodium borate, sodium chloride, and PURITE^®^ (stabilized oxychloro complex)
HPMC0.5	Sangthai Medical Co., Ltd., Bangkok, Thailand	0.5% Hydroxypropyl methyl cellulose	Benzalkonium chloride (other ingredients are not available.)
HYA0.1	Santen Pharmaceutical Co., Ltd., Osaka, Japan	0.1% Sodium hyaluronate (HYA)	ε-Aminocaproic acid, disodium edetate hydrate, propylene glycol, sodium chloride, chlorhexidine gluconate solution, and pH adjuster
HYA0.3	Santen Pharmaceutical Co., Ltd., Osaka, Japan	0.3% Sodium hyaluronate (HYA)	ε-Aminocaproic acid, disodium edetate hydrate, propylene glycol, sodium chloride, chlorhexidine gluconate solution, and pH adjuster
HPMC0.3S	Silom Medical Co., Ltd., Bangkok, Thailand	0.3% Hydroxypropyl methyl cellulose	Sodium perborate (other ingredients are not available.)

**Table 2:** Method validation data.

Parameters	Obtained Value ^a^	Criteria ^b^ [23,30,31]
Linearity		
Range	1–20 µg/mL	N/A
Slope	70,482	N/A
Y-intercept	2202	N/A
* r*	0.9998	≥0.9990
Precision		
Repeatability: %RSD	1.2	≤2.0
Intermediate: %RSD	2.0	≤2.0
Accuracy: %Recovery		
Solution	99.5 ± 1.2 ^c^ (1.2)	98–102%
Exemplified samples	99.1 ± 2.5 ^c^ (2.6)	95–105%
LOD	0.03	N/A
LOQ	0.10 (1.8)	N/A

^a^ Values in parentheses represent %RSDs; ^b^ N/A = not applicable; ^c^ Data are expressed as mean ± standard deviation (SD).

**Table 3:** Physical appearance and pH values of compatibility study between eight commercial artificial tears available in Thailand and ganciclovir solutions at the concentration of 5, 10, and 20 mg/mL.

Vehicles	pH before Drug Mixing	Concentration of Ganciclovir (mg/mL)					
		5		10		20	
		pH	Appearance ^a^	pH	Appearance ^a^	pH	Appearance ^a^
SWI	7.1	10.5	C	10.5	C	10.4	C
CMCG	7.3	7.9	C	8.4	P	9.2	P
DEXH	7.5	8.8	P*	9.2	P*	9.7	P*
PEGP	7.9	8.4	C	8.8	P	9.5	P
HPMC0.3A	6.7	8.3	C	8.8	C	9.4	P
CMC	7.2	8.3	C	8.7	P	9.4	P
HPMC0.5	7.0	9.3	P*	9.8	P*	10.2	P*
HYA0.1	6.3	9.9	C	10.1	C	10.3	C
HPMC0.3S	7.0	8.0	C	8.4	P	9.1	P

^a^ C = clear solution, P = precipitate occurs, and P* = precipitate occurs when stored in the refrigerator.

**Table 4:** Physicochemical and microbiological properties of 20 mg/mL ganciclovir/HYA0.1 and SWI EDs.

Tests	Ganciclovir/HYA0.1 ED	Ganciclovir/SWI ED
Appearance		
Color	Colorless	Colorless
Clarity	Clear	Clear
Precipitation	No	No
Gas bubble	No	No
pH	9.99 ± 0.08 ^a^	10.44 ± 0.06 ^a^
Osmolality (mOsm/kg H_2_O)	290 ± 1 ^a^	136 ± 1 ^a^
Viscosity (cP)	2.20 ± 0.01 ^a^	0.93 ± 0.06 ^a^
% Labeled amount	105.6 ± 6.6 ^a^	99.7 ± 0.7 ^a^
Sterility test	pass	pass

^a^ Data are expressed as mean ± SD.

**Table 5:** Appearance and sterility results of ganciclovir/HYA0.1 ED and ganciclovir/SWI ED after storage at 5 ± 3 °C for 24 weeks.

Test	Time (Weeks)											
	Ganciclovir/HYA0.1 ED						Ganciclovir/SWI ED					
	4	8	12	16	20	24	4	8	12	16	20	24
Packaging												
Integrity ^a^	C	C	C	C	C	C	C	C	C	C	C	C
Leakage ^b^	−	−	−	−	−	−	−	−	−	−	−	−
Solution												
Color ^c^	C	C	C	C	C	C	C	C	C	C	C	C
Clarity ^b^	+++	+++	+++	+++	+++	+++	+++	+++	+++	+++	+++	+++
Precipitation ^b^	−	−	+ ^d^	+ ^d^	+ ^d^	+ ^d^	−	−	−	−	−	−
Gas bubble ^b^	−	−	−	−	−	−	−	−	−	−	+++	+++
Sterility	Pass	Pass	Pass	Fail	ND ^e^	ND ^e^	Pass	Pass	Pass	Fail	ND ^e^	ND ^e^

^a^ C = completely sealed; ^b^ Scaling from − (could not be observed by the naked eye), +, ++, and +++ (clearly observable); ^c^ C = colorless; ^d^ Needle-like crystals were observed at the bottom of the bottle.; ^e^ ND = not determined.

**Table 6:** Appearance and sterility results of ganciclovir/HYA0.1 ED and ganciclovir/SWI ED after storage at 30 ± 2 °C for 12 weeks.

Test	Time (Weeks)					
	Ganciclovir/HYA0.1 ED			Ganciclovir/SWI ED		
	4	8	12	4	8	12
Packaging						
Integrity ^a^	C	C	C	C	C	C
Leakage ^b^	−	−	−	−	−	−
Solution						
Color ^c^	C	C	C	C	C	C
Clarity ^b^	+++	+++	++	+++	+++	++
Precipitation ^b^	−	−	−	−	−	−
Gas bubble ^b^	−	−	−	−	−	−
Sterility	Pass	Pass	Pass	Pass	Pass	Pass

^a^ C = completely sealed; ^b^ Scaling from − (could not be observed by the naked eye), +, ++, and +++ (clearly observable); ^c^ C = colorless.

**Table 7:** Stability results of ganciclovir/HYA0.3 ED after storage at 5 ± 3 °C and 30 ± 2 °C. The numerical results are expressed as mean (SD).

Test	Time (Weeks)							
	0	5 ± 3 °C						30 ± 2 °C
		4	8	12	16	20	24	4
Packaging								
Integrity ^a^	C	C	C	C	C	C	C	C
Leakage ^b^	−	−	−	−	−	−	−	−
Solution								
Color ^c^	C	C	C	C	C	C	C	C
Clarity ^b^	+++	+++	+++	+++	+++	+++	+++	+++
Precipitation ^b^	−	−	−	+ ^d^	+ ^d^	+ ^d^	+ ^d^	+
Gas bubble ^b^	−	−	−	−	−	−	−	−
pH ^e^	10.42 (0.02)	10.71 (0.03)	10.78 (0.07)	9.73 (0.00)	10.59 (0.00)	10.61 (0.06)	10.53 (0.06)	10.07 (0.03)
Viscosity ^e^ (cP)	6.69 (0.13)	6.59 (0.03)	6.68 (0.14)	6.41 (0.11)	6.51 (0.05)	6.28 (0.42)	6.52 (0.20)	6.12 (0.07)
Osmolality ^e^ (mOsm/kg H_2_O)	290 (2)	292 (1)	293 (1)	290 (0)	288 (2)	292 (1)	289 (1)	278 (1)
%Drug remaining ^e^	100.0 (3.4)	98.0 (3.9)	95.9 (4.8)	93.2 (7.7)	97.7 (4.3)	90.8 (9.9)	88.6 (2.0)	85.5 (3.0)

^a^ C = completely sealed; ^b^ Scaling from − (could not be observed by the naked eye), +, ++, and +++ (so much observable); ^c^ C = colorless; ^d^ Needle-like crystals were observed at the bottom of the bottle; ^e^ Values in parentheses represent SDs.

## Data Availability

The data presented in this study are available on request from the corresponding author.

## Supplementary Materials

The following supporting information can be downloaded at: https://www.mdpi.com/article/10.3390/pharmaceutics15010208/s1, Figure S1: Enlarged views of HPLC chromatograms of ganciclovir standard after being subjected to the stress conditions. (A) control; (B) acid hydrolysis; (C) base hydrolysis; (D) oxidation; (E) heat degradation and (F) photolytic decomposition.; Figure S2: HPLC chromatograms of ganciclovir after extraction of the spiked drug from ganciclovir/SWI, ganciclovir/HYA0.1, and ganciclovir/HYA0.3 EDs.; Figure S3: HPLC chromatograms of ganciclovir standard, ganciclovir/SWI ED, ganciclovir/HYA0.1 ED, and ganciclovir/HYA0.3 ED at the initial time (week 0), their shelf life (weeks 8 and 12), and the end of the study (weeks 24 and 12) when stored at 5 ± 3 °C (left panel) and at 30 ± 2 °C (right panel).

## References

[1] Miyazaki D., Shimizu D., Shimizu Y., Inoue Y., Inoue T., Higaki S., Ueta M., Sugita S., Miyazaki D., Shimizu D. (2018). Diagnostic efficacy of real-time PCR for ocular cytomegalovirus infections. Graefe’s Arch. Clin. Exp. Ophthalmol..

[2] Koizumi N., Miyazaki D., Inoue T., Ohtani F., Kandori-Inoue M., Inatomi T., Sotozono C., Nakagawa H., Horikiri T., Ueta M. (2017). The effect of topical application of 0.15% ganciclovir gel on cytomegalovirus corneal endotheliitis. Br. J. Ophthalmol..

[3] Koizumi N., Inatomi T., Suzuki T., Shiraishi A., Ohashi Y., Kandori M., Miyazaki D., Inoue Y., Soma T., Nishida K. (2015). Clinical features and management of cytomegalovirus corneal endotheliitis: Analysis of 106 cases from the Japan corneal endotheliitis study. Br. J. Ophthalmol..

[4] Chee S.-P., Bacsal K., Jap A., Se-Thoe S.-Y., Cheng C.L., Tan B.H. (2008). Clinical features of cytomegalovirus anterior uveitis in immunocompetent patients. Am. J. Ophthalmol..

[5] Koizumi N., Suzuki T., Uno T., Chihara H., Shiraishi A., Hara Y., Inatomi T., Sotozono C., Kawasaki S., Yamasaki K. (2008). Cytomegalovirus as an etiologic factor in corneal endotheliitis. Ophthalmology.

[6] Faith S.C., Durrani A.F., Jhanji V. (2018). Cytomegalovirus keratitis. Curr. Opin. Ophthalmol..

[7] Hwang Y.-S., Lin K.-K., Lee J.-S., Chang S.H.L., Chen K.-J., Lai C.-C., Huang J.C.-C., Kuo Y.-H., Hsiao C.-H. (2010). Intravitreal loading injection of ganciclovir with or without adjunctive oral valganciclovir for cytomegalovirus anterior uveitis. Graefe’s Arch. Clin. Exp. Ophthalmol..

[8] Villarreal E.C., Wu H., Lien E.J., Lien L.L., Schultz R.M., Ram V.J., Domingo E., Spence P., Gupta S.P., Bhat S.P., Villarreal E.C. (2003). Current and potential therapies for the treatment of herpesvirus infections. Progress in Drug Research.

[9] de Schryver I., Rozenberg F., Cassoux N., Michelson S., Kestelyn P., LeHoang P., Davis J.L., Bodaghi B. (2006). Diagnosis and treatment of cytomegalovirus iridocyclitis without retinal necrosis. Br. J. Ophthalmol..

[10] Crumpacker C.S. (1996). Ganciclovir. N. Engl. J. Med..

[11] Choi W.S., Cho J.H., Kim H.K., Kim H.S., Shin Y.J. (2013). A case of CMV endotheliitis treated with intravitreal ganciclovir injection. Korean J. Ophthalmol..

[12] Wong J.X.H., Agrawal R., Wong E.P.Y., Teoh S.C. (2016). Efficacy and safety of topical ganciclovir in the management of cytomegalovirus (CMV)-related anterior uveitis. J. Ophthalmic Inflamm. Infect..

[13] Fan N.-W., Chung Y.-C., Liu Y.-C., Liu C.J.-L., Kuo Y.-S., Lin P.-Y. (2016). Long-term topical ganciclovir and corticosteroids preserve corneal endothelial function in cytomegalovirus corneal endotheliitis. Cornea.

[14] Su C.-C., Wang I.J., Chen W.-L., Lin C.-P., His B., Hu F.-R. (2013). Topical ganciclovir treatment in patients with cytomegalovirus endotheliitis receiving penetrating keratoplasty. Clin. Experiment. Ophthalmol..

[15] Su C.-C., Hu F.-R., Wang T.-H., Huang J.-Y., Yeh P.-T., Lin C.-P., Wang I.J. (2014). Clinical outcomes in cytomegalovirus-positive Posner-Schlossman syndrome patients treated with topical ganciclovir therapy. Am. J. Ophthalmol..

[16] Keorochana N., Choontanom R. (2017). Efficacy and safety of an extemporaneous preparation of 2% ganciclovir eye drops in CMV anterior uveitis. BMJ Open Ophthalmol..

[17] Colin J. (2007). Ganciclovir ophthalmic gel, 0.15%: A valuable tool for treating ocular herpes. Clin. Ophthalmol..

[18] Srisangchun J., Noppawinyoowong C. (2013). Chemical stability and sterility of frozen ganciclovir injections. Srinagarind Med. J..

[19] Okumura N., Tanaka T., Fukui Y., Koizumi N. (2019). Stability, safety, and pharmacokinetics of ganciclovir eye drops prepared from ganciclovir for intravenous infusion. Jpn. J. Ophthalmol..

[20] Kathuria A., Shamloo K., Jhanji V., Sharma A. (2021). Categorization of marketed artificial tear formulations based on their ingredients: A rational approach for their use. J. Clin. Med..

[21] Brossard D., Chedru-Legros V., Crauste-Manciet S., Fleury-Souverain S., Lagarce F., Odou P., Roy S., Sadeghipour F., Sautou V. (2013). Methodological Guidelines for Stability Studies of Hospital Pharmaceutical Preparations Part 1: Liquid Preparations.

[22] (2003). ICH Topic Q 1 A (R2) Stability Testing of New Drug Substances and Products. https://www.ema.europa.eu/en/documents/scientific-guideline/ich-q-1-r2-stability-testing-new-drug-substances-products-step-5_en.pdf.

[23] (2022). United States Pharmacopeia 45-the National Formulary 40.

[24] (2005). ICH Harmonised Tripartite Guideline, Validation of Analytical Procedures: Text and Methodology Q2(R1). https://database.ich.org/sites/default/files/Q2%28R1%29%20Guideline.pdf.

[25] Ramesh P.J., Basavaiah K., Vinay K.B., Xavier C.M. (2012). Development and validation of RP-HPLC method for the determination of ganciclovir in bulk drug and in formulations. ISRN Chromatogr..

[26] Nuchtavorn N., Leanpolchareanchai J., Chanton D., Supapsophon P., Chongruchiroj S., Chatmapanrangsee J., Suksiriworapong J. (2021). A rapid stability indicating HPLC method for determination of quetiapine fumarate in tablets and extemporaneous formulations. Pharm. Chem. J..

[27] Doomkaew A., Prutthiwanasan B., Suntornsuk L. (2015). Stability indicating MEKC method for the determination of gliclazide and its specified impurities. J. Pharm. Biomed. Anal..

[28] Power L.A., Coyne J.W. (2018). ASHP guidelines on handling hazardous drugs. Am. J. Health Syst. Pharm..

[29] (2000). ICH Topic Q6A Specifications: Test Procedures and Acceptance Criteria for New Drug Substances and New Drug Products: Chemical Substances. https://www.ema.europa.eu/en/documents/scientific-guideline/ich-q-6-test-procedures-acceptance-criteria-new-drug-substances-new-drug-products-chemical_en.pdf.

[30] Epshtein N.A. (2020). System suitability requirements for liquid chromatography methods: Controlled parameters and their recommended values (review). Pharm. Chem. J..

[31] (1994). Center for Drug Evaluation and Research (CDER), Reviewer Guidance: Validation of Chromatographic Methods. https://www.fda.gov/media/75643/download.

[32] Guichard N., Bonnabry P., Rudaz S., Fleury-Souverain S. (2019). Long-term stability of ganciclovir in polypropylene containers at room temperature. J. Oncol. Pharm. Pract..

[33] Chen X., Ooi C.P., Lim T.H. (2006). Effect of ganciclovir on the hydrolytic degradation of poly(lactide-co-glycolide) microspheres. J. Biomater. Appl..

[34] (2019). Full Prescribing Information: Hialid 0.1 Ophthalmic Solution.

[35] Carney L.G., Mauger T.F., Hill R.M. (1989). Buffering in human tears: pH responses to acid and base challenge. Investig. Ophthalmol. Vis. Sci..

[36] Carney L.G., Hill R.M. (1979). Human tear buffering capacity. Arch. Ophthalmol..

[37] Bright A.M., Tighe B.J. (1993). The composition and interfacial properties of tears, tear substitutes and tear models. J. Br. Contact Lens Assoc..

[38] Willshire C., Buckley R.J., Bron A.J. (2018). Estimating basal tear osmolarity in normal and dry eye subjects. Cont. Lens Anterior Eye.

[39] Terry J.E., Hill R.M. (1978). Human tear osmotic pressure: Diurnal variations and the closed eye. Arch. Ophthalmol..

[40] Motolko M., Breslin C.W. (1981). The effect of pH and osmolarity on the ability of tolerate artificial tears. Am. J. Ophthalmol..

[41] Che Arif F., Hilmi M.R., Kamal K., Ithnin M. (2020). Evaluation of 18 artificial tears based on viscosity and pH. Malays. J. Ophthalmol..

[42] Simmons P.A., Liu H., Carlisle-Wilcox C., Vehige J.G. (2015). Efficacy and safety of two new formulations of artificial tears in subjects with dry eye disease: A 3-month, multicenter, active-controlled, randomized trial. Clin. Ophthalmol..

[43] Aragona P., Simmons P.A., Wang H., Wang T. (2019). Physicochemical properties of hyaluronic acid–based lubricant eye drops. Transl. Vis. Sci. Technol..

[44] Arshinoff S.A., Hofmann I., Nae H. (2021). Role of rheology in tears and artificial tears. J. Cataract Refract. Surg..

[45] Mole L., Oliva C., O’Hanley P. (1992). Extended stability of ganciclovir for outpatient parenteral therapy for cytomegalovirus retinitis. J. Acquir. Immune Defic. Syndr..

