# Supplementary material for: Extemporaneous Preparation of 20 mg/mL Ganciclovir in Artificial Tears in Comparison with Sterile Water for Ophthalmic Administration: Formulation and Stability Study
Source: Pharmaceutics. 2023 Jan 6;15(1):208. doi: 10.3390/pharmaceutics15010208 (PMC9866864; doi:10.3390/pharmaceutics15010208)
Supplement: Supplementary file 1 [file pharmaceutics-15-00208-s001.zip › pharmaceutics-2095911-supplementary.pdf]

Supplementary materials

Extemporaneous preparation of 2 mg/mL ganciclovir in artificial tears in comparison with sterile water for ophthalmic administration: Formulation and stability study

Jiraporn Leanpolchareanchai [1], Patamaporn Tangteerakoon [2], Patcharin Supapsophon [3], Somsiri Sukavatcharin [4], Pornchai Simaroj [4], Jiraphong Suksiriworapong [1,*]

[1] Department of Pharmacy, Faculty of Pharmacy, Mahidol University, Bangkok 10400, Thailand
[2] Chemotherapy Pharmacy Unit, Drug Compounding Section, Pharmacy Department, Faculty of Medicine, Ramathibodi Hospital, Bangkok 10400, Thailand
[3] Pharmacy Services, Somdech Phra Debaratana Medical Center, Faculty of Medicine, Ramathibodi Hospital, Bangkok 10400, Thailand
[4] Department of Ophthalmology, Faculty of Medicine, Ramathibodi Hospital, Mahidol University, Bangkok 10400, Thailand
* Correspondence: jiraphong.suk@mahidol.edu

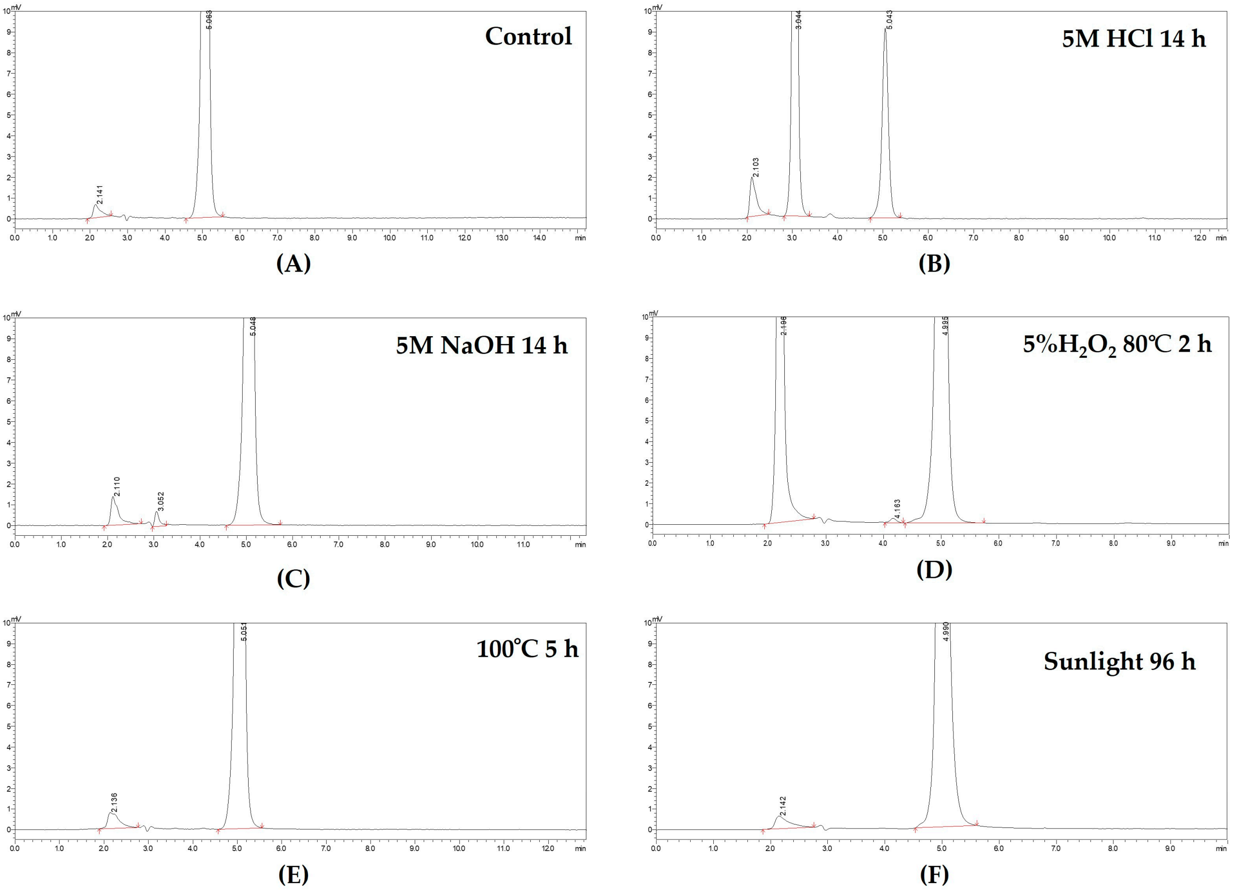

Figure S1. Enlarged views of HPLC chromatograms of ganciclovir standard after being subjected to the stress conditions. (A) control; (B) acid hydrolysis; (C) base hydrolysis; (D) oxidation; (E) heat degradation and (F) photolytic decomposition.

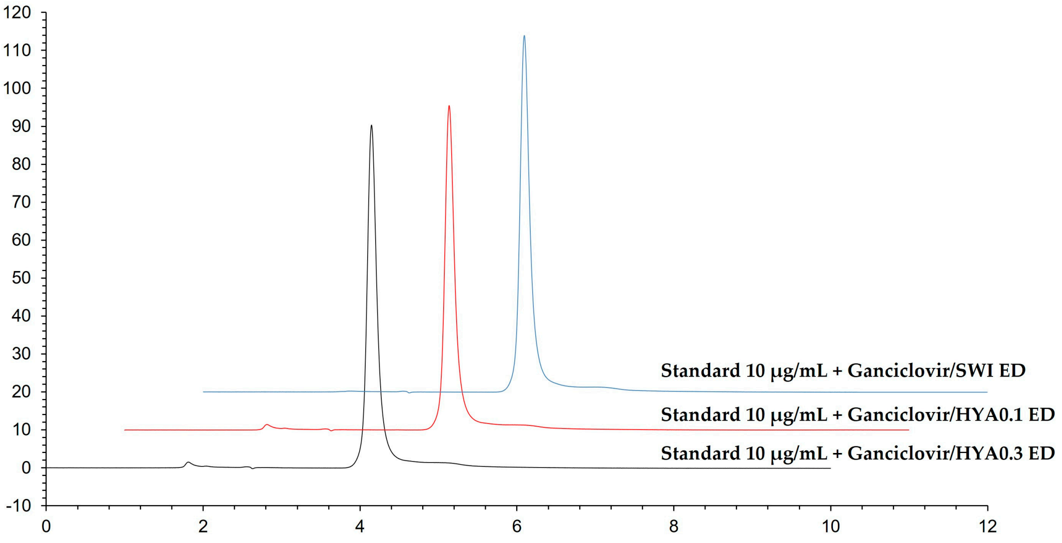

Figure S2. HPLC chromatograms of ganciclovir after extraction of the spiked drug from ganciclovir/SWI, ganciclovir/HYA0.1, and ganciclovir/HYA0.3 EDs.

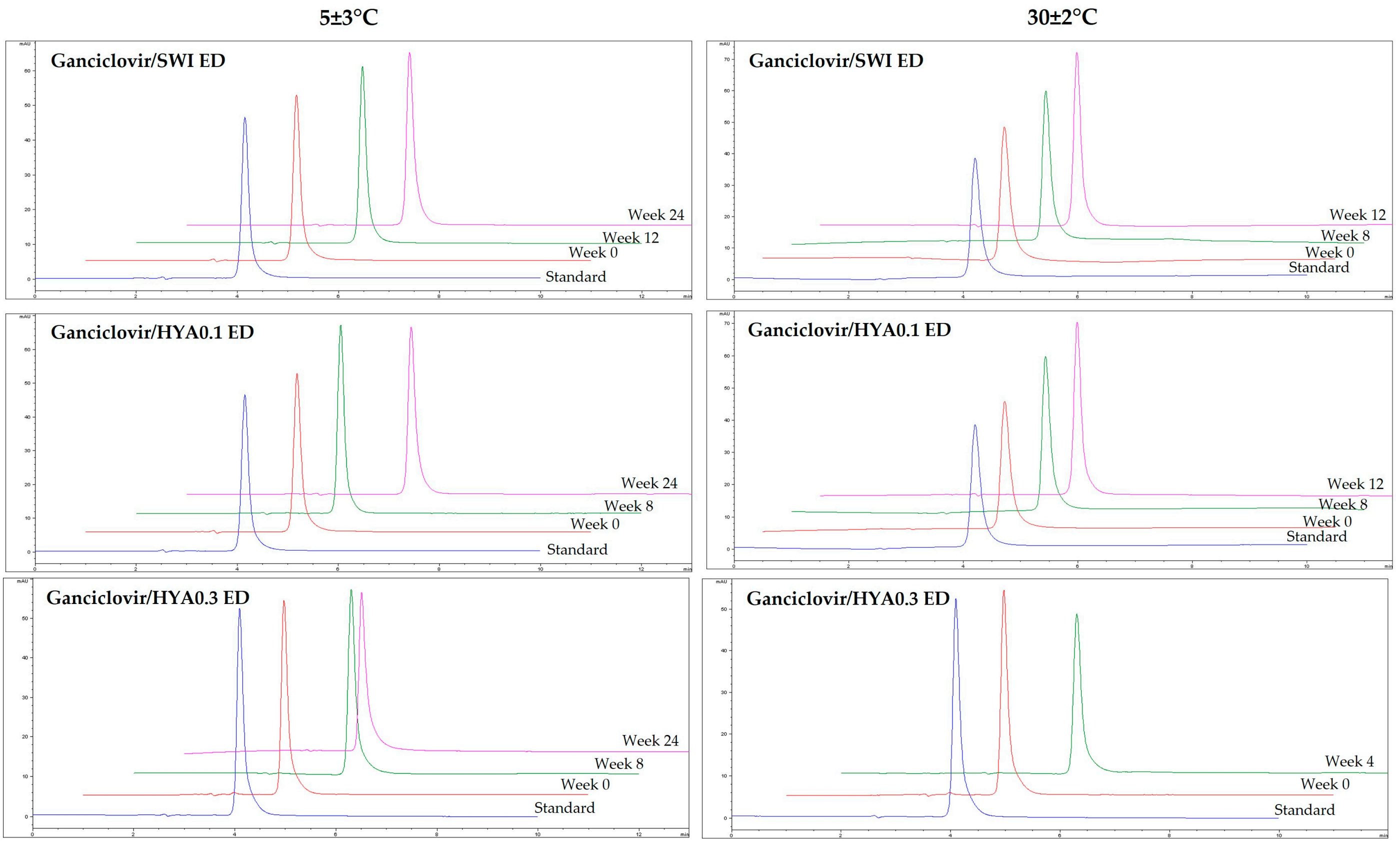

Figure S3. HPLC chromatograms of ganciclovir standard, ganciclovir/SWI ED, ganciclovir/HYA0.1 ED, and ganciclovir/HYA0.3 ED at the initial time (week 0), their shelf life (weeks 8 and 12), and the end of the study (weeks 24 and 12) when stored at 5 ± 3°C (left panel) and at 30 ± 2°C (right panel).
